# UK medical cannabis registry: A clinical outcome analysis of medical cannabis therapy in chronic pain patients with and without co‐morbid sleep impairment

**DOI:** 10.1111/papr.13438

**Published:** 2024-11-15

**Authors:** Ishita Datta, Simon Erridge, Carl Holvey, Ross Coomber, Rahul Guru, Wendy Holden, Alia Darweish Medniuk, Mohammed Sajad, Robert Searle, Azfer Usmani, Sanjay Varma, James J. Rucker, Michael Platt, Mikael H. Sodergren

**Affiliations:** ^1^ Department of Surgery and Cancer, Medical Cannabis Research Group Imperial College London London UK; ^2^ Curaleaf Clinic London UK; ^3^ St. George's Hospital NHS Trust London UK; ^4^ Cardiff and Vale University Health Board Cardiff UK; ^5^ Southmead Hospital North Bristol NHS Trust Bristol UK; ^6^ Department of Psychological Medicine Kings College London London UK; ^7^ South London and Maudsley NHS Foundation Trust London UK

**Keywords:** cannabinoids, CBD, chronic pain, medical cannabis, sleep impairment, THC

## Abstract

**Introduction:**

Chronic pain (CP) affects 35.0%–51.3% of the UK population, with 67%–88% reporting sleep disturbances. Cannabis‐based medicinal products (CBMPs) have shown therapeutic potential in managing CP. Evidence suggests poor sleep worsens pain perception; therefore, this study aimed to assess patient‐reported outcome measures (PROMs) following CBMP treatment in CP patients with and without co‐morbid sleep impairment.

**Methods:**

A prospective cohort study of CP patients from the UK Medical Cannabis Registry was conducted. Participants were separated by baseline single‐item sleep quality scale (SQS) score into sleep impaired (SQS ≤3) and unimpaired (SQS ≥4) cohorts. The primary outcome assessed changes in PROMs from baseline to 1‐, 3‐, 6‐, and 12‐months. Participants completed the following: SQS, General Anxiety Disorder‐7, EQ‐5D‐5L, Brief Pain Inventory (BPI), and Short‐Form McGill Pain Questionnaire‐2. Significance was defined as *p* < 0.050.

**Results:**

1139 participants met the inclusion criteria (sleep impaired: *n* = 517, 45.4%; sleep unimpaired: *n* = 622, 54.61%). The sleep impaired cohort showed improvements in all PROMs at each follow‐up (*p* < 0.010). The sleep unimpaired cohort showed similar results (*p* < 0.050), except in SQS and ED‐5Q‐5L: self‐care and anxiety/depression scores (*p* > 0.050). However, the sleep impaired cohort observed greater improvements in BPI pain severity (*p* < 0.050) and SQS (*p* < 0.001) than the sleep unimpaired cohort at all follow‐ups. 2817 adverse events were self‐reported between both cohorts (*p* = 0.197).

**Discussion:**

These findings align with literature that shows associated improvements in pain outcomes following CBMP administration. Sleep impaired individuals were more likely to experience greater pain severity improvements. However, this was not confirmed on multivariate logistic regression analysis and instead may be confounded by baseline pain severity.

**Conclusion:**

Whilst these results show promise for the effects of CBMPs on CP, they must be examined within the limitations of the study design. These findings provide further evidence to support the design of subsequent randomized controlled trials to verify causality between CBMPs and pain outcomes.

## INTRODUCTION

The treatment of chronic pain (CP) at present encompasses a range of treatments, including physiotherapy, psychotherapy, antidepressants, non‐steroidal anti‐inflammatory drugs (NSAIDs), opioids, and gabapentinoids.^1^ However, the evidence base for current pharmacotherapies typically suggests there is limited evidence of efficacy when used for CP,^2^, ^3^, ^4^, ^5^, ^6^ with emerging evidence suggesting they are also associated with significant adverse events.^7^, ^8^, ^9^, ^10^ Consequently, current treatment options are insufficient, as only 40% of individuals with CP report adequate pain relief.^11^ This highlights the need for more effective strategies to improve pain‐specific and health‐related quality‐of‐life (HRQoL) outcomes. Cannabis‐based medicinal products (CBMPs) have emerged as a potential therapeutic option to address this unmet need.^12^

Endocannabinoids and cannabinoid‐1 (CB1‐R) and cannabinoid‐2 (CB2‐R) receptors^13^ have been implicated in the pathophysiology of CP.^14^ Although both receptors are expressed throughout the nervous system, CB1‐Rs are predominantly expressed in the CNS, whilst CB2‐Rs are predominantly expressed on immune cells. The endocannabinoid system (ECS) plays a role in modulation of nociceptive stimuli, and the cognitive interpretation of pain at the peripheral, spinal, and supraspinal levels.^15^ Peripheral CB1‐R activation inhibits nociceptive transmission^16^ whilst central CB1‐R activation in the spinal dorsal horn inhibits pain neurotransmitter release,^17^ and in the thalamus inhibits ascending nociceptive transmission.^18^, ^19^ Conversely, CB2‐R activation increases β‐endorphins that act on μ‐opioid receptors to reduce pain signaling.^20^ The ECS extends to other receptors, including transient receptor potential vanilloid subtype‐1 (TRPV1), and 5‐hydroxytryptamine (5‐HT) receptors.^21^ TRPV1, located in the peripheral nervous system, mediates thermal hyperalgesia.^21^ Increased TRPV1 expression in dorsal root ganglia (DRG) has therefore been associated with long‐term thermal hyperalgesia, and mechanical allodynia.^22^ 5‐HT/5‐HT3A are found in over 70% of DRG,^23^ and are believed to contribute to the potentiation, and maintenance of TRPV1 sensitisation.^23^, ^24^

CBMPs derived from the cannabis plants contain over 144 active cannabinoids that interact with the ECS. The two most abundant compounds are (−)‐trans‐Δ^9^‐tetrahydrocannabinol (THC), and cannabidiol (CBD).^25^ THC is a partial agonist for CB1/2‐Rs^26^ Conversely, CBD inhibits the breakdown of the endogenous CB1‐R agonist, anandamide.^15^, ^27^ CBD has also been found to interact with TRVP1 signaling, whereby it inhibits adenyl cyclase to exert its analgesic effects.^28^

The evidence for CBMPs in CP management is mixed, with some studies, including previous UK Medical Cannabis Registry (UKMCR) studies^29^, ^30^, ^31^ showing associated improvements in pain‐specific and HRQoL outcomes following commencement of CBMPs,^32^, ^33^ and others finding a non‐significant difference.^34^ Discrepancies may arise due to methodological heterogeneity across primary literature, such as CBMP formulation differences, administration routes, and concentrations of constituent cannabinoids. However, a 2021 meta‐analysis found patients prescribed non‐inhaled CBMPs were 10% more likely to experience the minimum clinically important difference (MCID) in pain severity.^12^

Limited evidence exists regarding factors that influence which CP patients benefit most from CBMP treatment. Sleep quality is an important measure to consider, with sleep disturbance reported by 67%–88% of CP patients.^35^ The relationship between poor sleep and CP is bidirectional, whereby sleep impairment is both a cause and consequence of CP.^36^, ^37^ CP patients with sleep disturbances report longer sleep latency periods, fewer hours of total sleep, and poorer sleep quality, along with more severe and longer lasting pain, greater levels of mental distress, and poorer functioning.^38^ Therefore, CBMP treatment may help break this self‐perpetuating cycle to further improve patient outcomes.

Research has found cannabinoids can have positive and negative effects on sleep via the ECS. Anandamide plasma levels exhibit diurnal variation, suggesting their binding to CB1‐R induces sleep.^39^ Exogenous administration of anandamide has been demonstrated in pre‐clinical models to promote slow‐wave sleep and reduce wakefulness in a CB1‐R.^40^ The action of exogenous cannabinoids, such as CBD and THC, is therefore thought to replicate this activity. However, whilst a meta‐analysis of CBMPs used for CP found that they resulted in small improvements in subjective sleep quality, there is conflicting data for studies evaluating the efficacy of CBMPs as a treatment for insomnia.^12^, ^41^, ^42^ This is likely due to a paucity of high‐quality studies, which affects most literature exploring the therapeutic effects of CBMPs.

This study therefore primarily aimed to examine the relationship between CBMP treatment and patient‐reported outcome measures (PROMs) in CP patients with and without co‐morbid sleep impairment. Secondary aims included comparison of PROM score changes and AE incidence between each cohort and change in oral morphine equivalents (OMEs).

## METHODS

### Study design and participants

This prospective, observational cohort study utilized patient data from the UKMCR to evaluate efficacy, and safety of CBMP treatment for treating CP in patients with or without co‐morbid sleep impairment. The UKMCR, established by Curaleaf Clinic in 2019, is currently amongst the largest patient registries for prescribed CBMP use in Europe.^43^ Ethical approval was granted to the UKMCR by the Central Bristol Research Ethics Committee (reference: 22/SW/0145). The study adhered to the Strengthening the Reporting of Observational Studies in Epidemiology guidelines.^44^ All study participants provided written, and informed consent prior to consecutive enrolment.

The inclusion criteria required a primary diagnosis of CP refractory to conventional treatments,^1^ a minimum enrolment period of 1 year in the UKMCR at time of data extraction, and completion of baseline PROMs. A diagnosis of CP was determined by a consultant physician during the initial clinical consultation. Patients with a primary diagnosis of non‐CP conditions with secondary indications for CP treatment with CBMPs were excluded to prevent questionnaire fatigue.

The participants were classified into cohorts based on baseline single‐item sleep quality scale (SQS) score (Table 1): participants scoring ≤3 were allocated to the sleep impaired arm, and ≥4 to the sleep unimpaired arm.

**TABLE 1:** Descriptions of patient‐reported outcome measures (PROMs) collected from participants (*n* = 1139) at baseline and months 1, 3, 6, and 12.

Patient‐reported outcome measure		Description	Scoring
Pain‐specific	BPI	The BPI is a standardized, two‐part 11‐point scale^45^ that measures pain severity and its interference with activities of daily living, ranging from 0 (no pain/interference) to 10 (worst pain/complete interference).^46^ The MCID in BPI pain severity corresponds to a decrease of ≥1.^47^	0–10
	SF‐MPQ‐2	The SF‐MPQ‐2 is an 11‐point scale with 22 descriptors^45^ that cover 4 overarching domains: continuous, intermittent, neuropathic and affective pain. Each descriptor is rated 0–10, where a score of 0 indicates no pain and 10 indicates worst pain.^48^ A mean score is generated for each domain, as well as an overall pain score.^49^	0–10
Health‐related quality of life	GAD‐7	The GAD‐7 is a 21‐point numerical rating scale used to measure anxiety severity by rating the frequency of 7 symptoms from 0 (not at all) to 3 (nearly every day) over the past 2 weeks, resulting in a total score range of 0–21.^50^, ^51^	0–21
	SQS	The SQS requires patients to rate their overall sleep quality from 0 to 10 over the past 7 days. The scale is divided into 5 categories from ‘terrible’ to ‘excellent’ based on the score.^52^ An increase in SQS of ≥2.6 is determined as clinically significant.^52^	0–10
	EQ‐5D‐5L	The EQ‐5D‐5L is comprised of 5 domains: mobility, self‐care, usual activities, pain/discomfort and anxiety/depression, and each domain is rated on a 5‐point Likert scale from ‘no problems’ to ‘extreme problems’.^53^ The digits from the 5 domains can be combined and translated into an index value ranging from 1 (full health) to <0 (health status worse than death).^54^	<0–1
	PGIC	The PGIC is a 7‐point NRS that assesses the participant's perception of improvement compared to their baseline after treatment initiation,^55^ where 0 represents ‘no change’ and 7 represents ‘considerable improvement’.^56^	0–7

Abbreviations: BPI, brief pain inventor; SF‐MPQ‐2, short‐form McGill pain questionnaire‐2; GAD‐7, generalized anxiety disorder‐7; SQS, single‐item sleep quality scale; EQ‐5D‐5L, European quality‐of‐life five dimensions five levels; PGIC, patients' global impression of change.

### Data collection

Upon enrolment in the UKMCR, baseline questionnaires captured demographic data, including age, gender, occupation, body mass index (BMI), co‐morbidities, tobacco history, alcohol consumption, and prior cannabis consumption. Incomplete fields were reviewed and updated by a member of the clinical and/or research team by contacting the patient after initial consultation. The Charlson‐comorbidity index, a validated diagnostic and prognostic tool^57^, ^58^ used in other registries to quantify co‐morbidity data,^58^ was calculated for each patient based on their inputted medical history. Tobacco use, smoking pack‐years, and weekly alcohol consumption were also recorded. Prior to CBMP prescription, patients' cannabis history was obtained and classified into three categories: cannabis‐naïve users (no prior cannabis use), ex‐users (previous cannabis use but not using at the time of enrolment), and current users (cannabis use up until time of prescription). To quantify cannabis use in ex‐ and current cannabis users, a novel metric known as cannabis gram years was employed. This measure has been used in past UKMCR studies^30^, ^31^, ^59^ and is similar to the validated measure of smoking pack‐years for defining tobacco cigarette use.^60^

Data on CBMP prescription, including formulation, dosage, administration route, and cannabinoid contents, were collected and documented each time medication was dispensed to the patient.

Self‐reporting via PROMs is considered the benchmark for evaluating CP conditions.^45^, ^61^ Baseline PROMs were distributed to all patients upon registration, with data collected electronically from patients or entered by clinicians during initial clinical consultations.^62^ These PROMs were repeated at 1‐, 3‐, 6‐, and 12‐months, with patients prompted every 72 h until completion.

### Patient‐related outcome measures

PROMs collected in this study were categorized into pain‐specific, and HRQoL PROMs (Table 1).^45^, ^46^, ^48^, ^49^, ^50^, ^51^, ^52^, ^53^, ^54^, ^55^, ^56^

### Missing data

To account for missing PROM data due to incompletion or patient dropout, a baseline observation carried forward approach was utilized to account for loss to follow up in a conservative manner, and prevent overstatement of findings.^63^ This involved replacing missing fields with participants' baseline PROM scores, as it was assumed they would return to baseline values after CBMP treatment cessation.

### Opiate prescriptions

Patients receiving opioid prescriptions at any point during the study were identified. Daily oral morphine equivalent (OME) doses (mg/day) were calculated using the British National Formulary and Royal College of Anesthetists conversion factors at baseline and at each follow‐up timepoint.

### Adverse events

Patients self‐reported any AEs contemporaneously with PROMs, electronically when they occurred, or during clinical consultations if otherwise still unreported. These were recorded in accordance with the Common Terminology Criteria for Adverse Events version 4.0.^64^

### Outcomes

The primary outcomes of this study were to analyze changes in PROM scores from baseline to 1‐, 3‐, 6‐, and 12‐month follow‐ups. Secondary outcomes were to analyze differences in PROM score changes and AE incidence, and severity between the two cohorts with impaired, and unimpaired sleep.

### Statistical analysis

Descriptive statistics were used to display demographic variables, PROM scores, and AE frequencies using mean ± standard deviation (SD) for parametric data and median ± interquartile range (IQR) for non‐parametric data. Subsequently, inferential statistics were employed to identify differences between cohorts and changes in follow‐up PROM scores compared to baseline. Statistical significance was defined as *p* < 0.050 for all tests performed.

Categorical data was analyzed using Chi‐squared tests, whilst parametric and non‐parametric data were analyzed using two‐tailed independent *t*‐tests and Mann Whitney‐*U* rank‐sum tests, respectively. PROM data was treated as parametric due to the central limit theorem.^65^ For longitudinal analysis, such as comparisons of PROM scores at follow‐up months 1‐, 3‐, 6‐, and 12‐ against baseline, a repeated measures analysis of variance (ANOVA) was employed to minimize type I error risk.^66^ Post‐hoc pairwise comparison of statistically significant values on the repeated measures ANOVA underwent Bonferroni correction to further limit type I error.^66^, ^67^ Additionally, independent *t*‐tests were performed to analyze differences in the percentage change in PROM scores from baseline to follow‐ups between the cohorts.

Univariate and multivariate logistic regression analyses were conducted to assess the associated odds ratios (ORs) with relevant co‐variates and the likelihood of experiencing the MCID in pain severity at 12‐months or one or more adverse events at any period during follow up.

All statistical analyses were carried out using Statistical Package for the Social Sciences (SPSS; v.29.0.0.0), and graphs were created using GraphPad Prism (v. 9.4.1(350)).

## RESULTS

**FIGURE 1:**
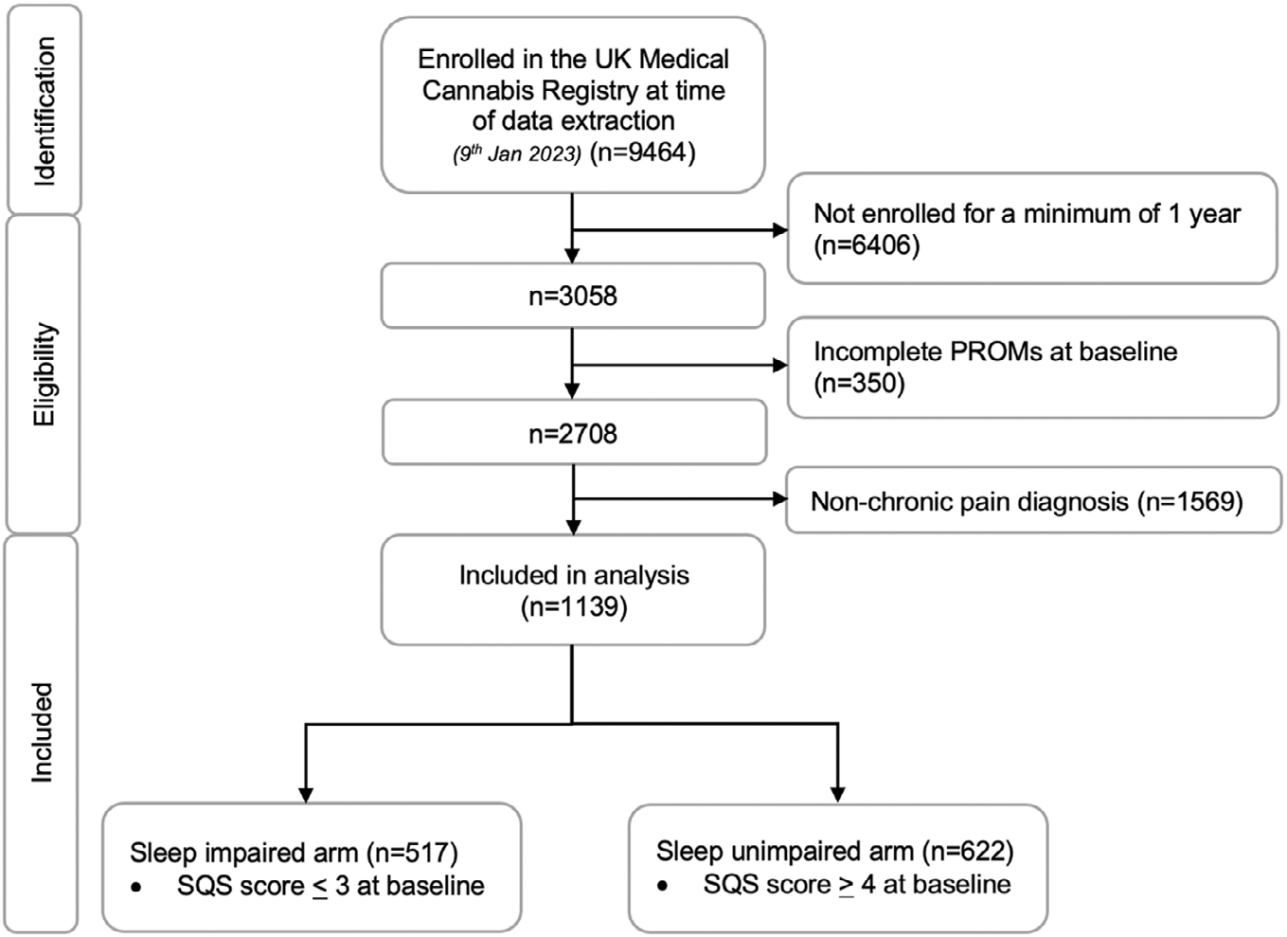
Flow diagram depicting identification, eligibility and exclusion criteria for patients enrolled in the UK Medical Cannabis Registry at time of data extraction (*n* = 9464). The number and reasons for excluding participants are illustrated in a stepwise manner leaving 1139 patients included in the analysis. This is divided into the two cohorts based on their sleep quality scale scores: Sleep impaired (*n* = 517) and sleep unimpaired (*n* = 622).

The UKMCR had 9464 patients enrolled at the time of data extraction (9th January 2023). Patients were excluded if they had not been enrolled for at least 1 year (*n* = 6404, 67.7%), had incomplete baseline PROMs data (*n* = 350, 3.7%), or had a non‐CP primary diagnosis (*n* = 1569, 16.6%). This resulted in a sample of 1139 patients (Figure 1). The sample was then divided into two cohorts based on baseline single‐item SQS score: patients with a score of ≤3 were assigned to the sleep impaired arm (*n* = 517, 45.4%), whilst patients with a score of ≥4 were assigned to the sleep unimpaired arm (*n* = 622, 54.6%).

### Baseline demographics

The baseline demographic details for both cohorts are displayed in Table 2. The female‐to‐male ratio was lower in the sleep impaired cohort (0.75:1) than the sleep unimpaired cohort (1:1; *p* = 0.013). Employment status differed between the cohorts (*p* < 0.001), with the sleep impaired arm predominantly unemployed (*n* = 274, 53.0%) whereas the sleep unimpaired arm predominantly employed (*n* = 340, 54.7%). The most common indication for CBMP treatment in both cohorts was ‘chronic non‐cancer pain’, followed by ‘neuropathic pain’. The IQR for the Charlson co‐morbidity index was wider in the sleep impaired (1.0 [0.0–6.0]) than unimpaired arm (1.0 [0.0–5.0]; *p* = 0.018), with the individual co‐morbidity incidences displayed in Appendix A (Supporting Information).

**TABLE 2:** Baseline demographic details of study participants (*n* = 1139) at baseline assessment. The occupation category ‘unemployed’ also includes those who were retired, and the occupation category ‘undefined’ includes students, under‐18 s, and those who had occupation data missing. Number of participants (*n*) missing data is indicated with a separate key in the footnotes.

Baseline demographics	Sleep impaired (*n* = 517)	Sleep unimpaired (*n* = 622)	*p*‐value
	n (%) / mean ± SD/median [IQR]		
Gender			
Male	258 (49.9%)	356 (57.2%)	0.013*
Female	259 (50.1%)	266 (42.8%)	
Age (years)	47.0 ± 14.1	45.7 ± 14.7	0.125
^a^ Body mass index (kg/m^2^)	27.9 ± 7.4	27.1 ± 6.4	0.067
Occupation			
Employed	220 (42.6%)	340 (54.7%)	<0.001***
Unemployed	274 (53.0%)	244 (39.2%)	
Undefined	23 (4.4%)	38 (6.1%)	
Pain etiology			
Cancer pain	0 (0.0%)	2 (0.3%)	0.314
Chronic non‐cancer pain	290 (56.1%)	377 (60.6%)	
Complex regional pain syndrome	5 (1.0%)	8 (1.3%)	
Ehlers‐Danlos	32 (6.2%)	43 (6.9%)	
Inflammatory arthritis	40 (7.7%)	42 (6.8%)	
Neuropathic pain	108 (20.9%)	115 (18.5%)	
Osteoarthritis	42 (8.1%)	35 (5.6%)	
Charlson co‐morbidity index	1.0 [0.0–6.0]	1.0 [0.0–5.0]	0.018*

*Note*: Statistical analysis by chi‐squared for categorical data, independent *t*‐test for parametric data and Mann–Whitney *U* for non‐parametric data with significant differences between the arms denoted as *p < 0.050, **p < 0.010, ***p < 0.001.

Abbreviations: IQR, interquartile range; *N*, number of participants; SD, standard deviation.

^a^ Missing data: sleep impaired (*n* = 41, 7.9%); sleep unimpaired (*n* = 52, 8.4%).

Tobacco, alcohol, and cannabis consumption for both cohorts are displayed in Table 3. Tobacco status differed between the groups (*p* = 0.030), with the sleep unimpaired group having a higher proportion of non‐smokers and ex‐smokers. Although there was no difference in baseline cannabis status between the cohorts (*p* = 0.280), the sleep impaired arm had greater median cannabis gram years (8.0 [2.0–20.0]) at baseline than the sleep unimpaired arm (6.0 [1.9–18.0]; *p* = 0.027).

**TABLE 3:** Baseline demographic details of study participants (*n* = 1139) at baseline assessment. The occupation category ‘unemployed’ also includes those who were retired, and the occupation category ‘undefined’ includes students, under‐18 s, and those who had occupation data missing. Number of participants (*n*) missing data is indicated with a separate key in the footnotes.

Tobacco, alcohol and cannabis consumption	Sleep impaired (*n* = 517)	Sleep unimpaired (*n* = 622)	*p*‐value
	*n* (%)/median [IQR]		
Tobacco status			
Current smoker	161 (31.1%)	150 (24.1%)	0.030*
Ex‐smoker	204 (39.5%)	268 (43.1%)	
Non‐smoker	152 (29.4%)	204 (32.8%)	
^a^ Smoking pack years	10.0 [3.0–20.0]	10.0 [4.0–20.0]	0.317
^b^ Weekly alcohol consumption (units)	0.0 [0.0–4.0]	0.0 [0.0–5.0]	0.081
Cannabis status			
Current user	263 (50.9%)	345 (55.5%)	0.280
Ex‐user	76 (14.7%)	89 (14.3%)	
Cannabis naïve	178 (34.4%)	188 (30.2%)	
^c^ Cannabis gram years	8.0 [2.0–20.0]	6.0 [1.9–18.0]	0.027*
Frequency of cannabis use			
Every day	224 (43.3%)	296 (47.6%)	0.661
Every other day	20 (3.9%)	25 (4.1%)	
1–2 times per week	12 (2.3%)	18 (2.9%)	
>1 times per month	3 (0.6%)	2 (0.3%)	
<1 times per month	4 (0.8%)	4 (0.6%)	
Undefined	254 (49.1%)	277 (44.5%)	

*Note*: Statistical analysis by Chi‐squared for categorical data, independent *t*‐test for parametric data and Mann–Whitney *U* for non‐parametric data with significant differences between the arms denoted as *p < 0.050, **p < 0.010, ***p < 0.001.

Abbreviations: IQR, interquartile range; *N*, number of participants; SD, Standard deviation.

^a^ Missing data: Sleep impaired (*n* = 142, 27.5%), sleep unimpaired (*n* = 189, 30.4%).

^b^ Missing data: Sleep impaired (*n* = 4, 0.8%), sleep unimpaired (*n* = 1, 0.2%).

^c^ Missing data: Sleep impaired (*n* = 339, 34.4%), sleep unimpaired (*n* = 188, 30.2%).

### CBMP treatment details

CBMP treatment details of the maximally titrated dose for both cohorts is displayed in Table 4. The most common formulations were Adven®20 and Adven®50 sublingual medium‐chain triglyceride oils and Adven®EMT1 vapourised dry flower (Curaleaf International, Guernsey, UK). A combination of CBD and THC was prescribed to most participants in the sleep impaired (*n* = 493, 95.7%) and unimpaired cohort (*n* = 594, 96.3%; *p* = 0.892), with no difference between doses (CBD: 22.5 [20.0–40.0] mg/day and 20.0 [15.8–40.0] mg/day, respectively, *p* = 0.446; THC: 112.5 [143.1–214.5] mg/day and 111.6 [14.6–214.8] mg/day, respectively; *p* = 0.672).

**TABLE 4:** Details of cannabis‐based medicinal products prescribed at maximally titrated dose for study participants (*n* = 1132).

CBMP details	Sleep impaired (*n* = 515)	Sleep unimpaired (*n* = 617)	*p*‐value
	*n* (%)/median [IQR]		
Cannabinoid contents			
No. of patients on CBD only	7 (1.4%)	7 (1.1%)	0.892
No. of patients on THC only	15 (2.951%)	16 (2.6%)	
No. of patients on CBD and THC	493 (95.7%)	594 (96.3%)	
^a^ Administration routes			
No. of patients on sublingual/oral formulations only	150 (29.1%)	192 (31.1%)	0.116
No. of patients on vaporized flower only	104 (20.2%)	149 (24.1%)	
No. of patients on both	261 (50.7%)	277 (44.8%)	
Dosage			
CBD dosage (mg/day)	22.5 [20.0–40.0]	20.0 [15.8–40.0]	0.446
THC dosage (mg/day)	112.5 [143.1–214.5]	111.6 [14.6–214.8]	0.672

*Note*: Statistical analysis by Chi‐squared for categorical data and Mann–Whitney *U* for non‐parametric data with significant differences between the sleep impaired (*n* = 515), and sleep unimpaired (*n* = 617) arms denoted as *p < 0.050, **p < 0.010, ***p < 0.001.

Abbreviations: CBD, Cannabidiol; IQR, interquartile range; mg/day, milligrams per day; *N*, number of participants; THC, ∆^9^‐tetrahydrocannabinol.

^a^ Sleep unimpaired (*n* = 618).

### Patient‐reported outcome measures

Differences in baseline PROM scores between the cohorts are displayed in Appendix B (Supporting Information), with it showing the sleep impaired arm had worse baseline scores than the sleep unimpaired arm across all PROMs (*p* < 0.050). The mean ± SD of pain‐specific and HRQoL PROM scores at baseline, and follow‐up (1‐, 3‐, 6‐, and 12‐months) are presented in Table 5, with mean percentage changes from baseline outlined in Appendix C (Supporting Information). Improvements from baseline were observed in all PROM follow‐ups in the sleep impaired cohort (*p* < 0.010), and in most in the sleep unimpaired cohort (*p* < 0.050) except at SQS follow‐up months 1 and 12, all EQ‐5D‐5L self‐care follow‐ups, and EQ‐5D‐5L anxiety/depression follow‐up month 12 (*p* > 0.050).

**TABLE 5:** Paired mean ± standard deviation patient‐reported outcome measure (PROM) scores at baseline and follow‐up months 1, 3, 6, and 12 of the sleep impaired (*n* = 517), and unimpaired (*n* = 622) arms. Baseline PROM defined at month 0.

Patient‐reported outcome measure		Month	Sleep impaired (*n* = 517)		Sleep unimpaired (*n* = 622)	
			Mean score ± SD	*p*‐value	Mean score ± SD	*p*‐value
Pain‐specific	BPI: pain severity	0	6.44 ± 1.62	–	5.26 ± 1.75	–
		1	5.70 ± 1.87	<0.001***	4.84 ± 1.85	<0.001***
		3	5.60 ± 1.88	<0.001***	4.70 ± 1.96	<0.001***
		6	5.67 ± 2.00	<0.001***	4.77 ± 1.94	<0.001***
		12	5.86 ± 1.91	<0.001***	4.94 ± 1.89	<0.001***
	BPI: pain interference	0	7.54 ± 1.86	–	5.63 ± 2.25	–
		1	6.36 ± 2.34	<0.001***	4.99 ± 2.41	<0.001***
		3	6.16 ± 2.40	<0.001***	4.82 ± 2.52	<0.001***
		6	6.37 ± 2.46	<0.001***	4.96 ± 2.46	<0.001***
		12	6.79 ± 2.32	<0.001***	5.18 ± 2.44	<0.001***
	SF‐MPQ‐2	0	5.28 ± 1.97	–	3.82 ± 1.91	–
		1	4.50 ± 2.18	<0.001***	3.39 ± 1.92	<0.001***
		3	4.50 ± 2.24	<0.001***	3.26 ± 2.02	<0.001***
		6	4.48 ± 2.20	<0.001***	3.28 ± 2.00	<0.001***
		12	4.74 ± 2.17	<0.001***	3.43 ± 2.01	<0.001***
Health‐related quality of life	GAD‐7	0	9.19 ± 6.48	–	5.36 ± 5.23	–
		1	6.76 ± 6.06	<0.001***	4.31 ± 4.38	<0.001***
		3	7.16 ± 6.10	<0.001***	4.59 ± 4.92	<0.001***
		6	7.42 ± 6.17	<0.001***	4.56 ± 4.84	<0.001***
		12	7.95 ± 6.21	<0.001***	4.88 ± 5.03	0.005**
	SQS	0	1.99 ± 1.09	–	6.05 ± 1.70	–
		1	4.26 ± 2.50	<0.001***	6.28 ± 2.10	0.059
		3	4.02 ± 2.58	<0.001***	6.36 ± 2.11	0.001**
		6	3.81 ± 2.57	<0.001***	6.28 ± 2.01	0.017*
		12	3.37 ± 2.34	<0.001***	6.20 ± 1.97	0.257
	EQ‐5D‐5L: mobility	0	3.13 ± 1.05	–	2.58 ± 1.16	–
		1	2.86 ± 1.11	<0.001***	2.47 ± 1.09	<0.001***
		3	2.87 ± 1.12	<0.001***	2.46 ± 1.41	<0.001***
		6	2.89 ± 1.14	<0.001***	2.43 ± 1.14	<0.001***
		12	2.97 ± 1.11	<0.001***	2.51 ± 1.15	0.012*
	EQ‐5D‐5L: self‐care	0	2.41 ± 1.12	–	1.89 ± 1.00	–
		1	2.26 ± 1.09	<0.001***	1.88 ± 1.01	1.000
		3	2.25 ± 1.10	<0.001***	1.86 ± 0.99	1.000
		6	2.28 ± 1.09	<0.001***	1.82 ± 0.98	0.114
		12	2.31 ± 1.10	0.001**	1.85 ± 0.98	0.751
	EQ‐5D‐5L: usual activities	0	3.33 ± 1.08	–	2.70 ± 1.13	–
		1	2.90 ± 1.08	<0.001***	2.41 ± 1.04	<0.001***
		3	2.97 ± 1.11	<0.001***	2.46 ± 1.13	<0.001***
		6	2.96 ± 1.12	<0.001***	2.46 ± 1.10	<0.001***
		12	3.11 ± 1.07	<0.001***	2.55 ± 1.10	<0.001***
	EQ‐5D‐5L: pain/discomfort	0	4.06 ± 0.83	–	3.39 ± 0.91	–
		1	3.43 ± 0.97	<0.001***	3.00 ± 0.92	<0.001***
		3	3.47 ± 1.00	<0.001***	2.98 ± 0.97	<0.001***
		6	3.53 ± 0.99	<0.001***	3.02 ± 0.96	<0.001***
		12	3.62 ± 1.01	<0.001***	3.15 ± 0.96	<0.001***
	EQ‐5D‐5L: anxiety/depression	0	2.71 ± 1.23	–	2.02 ± 1.05	–
		1	2.34 ± 1.16	<0.001***	1.86 ± 0.96	<0.001***
		3	2.41 ± 1.17	<0.001***	1.92 ± 0.98	0.010*
		6	2.48 ± 1.19	<0.001***	1.92 ± 1.00	0.008**
		12	2.53 ± 1.20	<0.001***	1.97 ± 1.01	0.480
	EQ‐5D‐5L: index value	0	0.21 ± 0.30	–	0.45 ± 0.28	–
		1	0.38 ± 0.31	<0.001***	0.53 ± 0.26	<0.001***
		3	0.36 ± 0.32	<0.001***	0.52 ± 0.27	<0.001***
		6	0.35 ± 0.32	<0.001***	0.52 ± 0.27	<0.001***
		12	0.31 ± 0.32	<0.001***	0.50 ± 0.27	<0.001***
	^†^PGIC	0	–	–	–	–
		1	4.79 ± 1.58	–	5.16 ± 1.57	–
		3	5.02 ± 1.54	–	5.29 ± 1.54	–
		6	5.15 ± 1.47	–	5.39 ± 1.48	–
		12	5.20 ± 1.54	–	5.39 ± 1.51	–

*Note*: Statistical analysis using repeated measures analysis of variance for comparison of follow‐up scores against baseline score, with significant differences denotes as *p < 0.050, **p < 0.010, ***p < 0.001.

Abbreviations: BPI, Brief pain inventory; GAD‐7, Generalized anxiety disorder‐7; N, Number of participants; PGIC, Patients' global impression of change; SD, Standard deviation; SF‐MPQ‐2, Short‐form McGill pain questionnaire‐2; SQS, Single‐item sleep quality scale.

The mean percentage change in PROM scores at each follow‐up from baseline for the cohorts are displayed and compared in Table 6, with raw mean differences in PROM scores displayed in Appendix D (Supporting Information). Most notably, the sleep impaired cohort consistently showed greater improvements than the sleep unimpaired cohort in BPI pain severity, SQS, and EQ‐5D‐5L: pain/discomfort and anxiety/depression at all follow‐ups (*p* < 0.050). According to mean PGIC scores displayed in Appendix E (Supporting Information), both cohorts felt they had improved in their overall quality of life; however, the sleep impaired cohort had a higher perceived improvement (*p* < 0.050).

**TABLE 6:** Mean percentage change ± standard deviation in patient‐reported outcome measure (PROM) scores at follow‐up months 1, 3, 6, and 12 compared to baseline.

Patient‐reported outcome measures		Month	Sleep impaired		Sleep unimpaired		*p*‐value
			*n*	Mean change in score ± SD (%)	*n*	Mean change in score ± SD (%)	
Pain‐specific	BPI: pain severity	1	517	−10.33 ± 24.03	622	−3.28 ± 41.77	<0.001***
		3	517	−11.91 ± 24.79	622	−7.19 ± 38.87	0.013*
		6	517	−11.52 ± 24.29	622	−6.58 ± 39.84	0.014*
		12	517	−8.90 ± 21.00	622	−3.34 ± 35.30	0.002**
	BPI: pain interference	1	517	−15.23 ± 26.73	622	−1.61 ± 132.20	0.013*
		3	517	−17.59 ± 29.85	622	−6.52 ± 112.62	0.019*
		6	517	−15.24 ± 28.32	622	−2.42 ± 158.91	0.070
		12	517	−9.53 ± 26.03	622	−2.00 ± 142.51	0.070
	SF‐MPQ‐2	1	517	−14.63 ± 30.22	620	−0.62 ± 150.58	0.024*
		3	517	−14.32 ± 31.28	620	−2.99 ± 212.77	0.230
		6	517	−14.26 ± 31.82	620	−2.11 ± 207.38	0.188
		12	516	−9.83 ± 29.19	620	−0.08 ± 171.95	0.203
Health‐related quality of life	GAD‐7	1	506	−17.58 ± 67.85	589	−2.90 ± 106.86	0.008**
		3	506	−12.96 ± 66.45	597	−1.40 ± 95.88	0.022*
		6	505	−9.94 ± 75.59	600	−3.70 ± 74.84	0.169
		12	507	−2.95 ± 101.19	603	−3.01 ± 94.15	0.310
	Single‐item SQS	1	460	108.01 ± 150.24	622	7.90 ± 40.00	<0.001***
		3	469	91.12 ± 147.24	622	8.75 ± 38.45	<0.001***
		6	472	78.39 ± 138.13	622	7.46 ± 35.95	<0.001***
		12	483	56.66 ± 115.14	622	5.18 ± 30.83	<0.001***
	EQ‐5D‐5L: mobility	1	517	−5.73 ± 33.12	622	0.86 ± 35.57	0.001**
		3	517	−5.46 ± 32.55	622	−0.61 ± 33.83	0.014*
		6	517	−5.72 ± 29.35	622	−1.62 ± 31.77	0.025*
		12	517	−2.93 ± 29.09	622	0.38 ± 28.61	0.053
	EQ‐5D‐5L: self‐care	1	517	0.02 ± 37.62	622	5.51 ± 41.47	0.020*
		3	517	−1.37 ± 36.20	622	3.72 ± 35.52	0.017*
		6	517	−0.27 ± 34.86	622	1.87 ± 35.16	0.304
		12	517	0.38 ± 36.05	622	2.14 ± 30.24	0.371
	EQ‐5D‐5L: usual activities	1	517	−7.30 ± 44.26	622	−4.32 ± 37.71	0.219
		3	517	−6.30 ± 38.62	622	−2.70 ± 42.68	0.139
		6	517	−7.23 ± 37.92	622	−4.36 ± 31.86	0.165
		12	517	−3.31 ± 31.80	622	−0.95 ± 35.65	0.243
	EQ‐5D‐5L: pain/discomfort	1	517	−13.28 ± 25.51	622	−8.55 ± 27.92	0.002**
		3	517	−12.03 ± 20.70	622	−9.54 ± 26.30	0.010*
		6	517	−9.88 ± 20.30	622	−9.31 ± 22.01	0.033*
		12	516	−8.42 ± 35.52	622	−5.43 ± 21.21	<0.001***
	EQ‐5D‐5L: anxiety/depression	1	517	−8.42 ± 35.52	621	−1.23 ± 36.66	<0.001***
		3	517	−4.63 ± 40.50	621	2.95 ± 44.40	0.003**
		6	517	−3.24 ± 36.61	621	1.66 ± 38.50	0.029*
		12	517	−2.87 ± 29.07	621	3.44 ± 37.96	0.002**
	EQ‐5D‐5L: index value	1	517	547.81 ± 3379.27	621	219.87 ± 2621.07	0.072
		3	517	285.74 ± 2265.85	621	106.12 ± 534.13	0.079
		6	517	400.39 ± 2828.22	621	108.72 ± 732.09	0.023*
		12	517	267.57 ± 1911.23	621	82.39 ± 649.65	0.036*

*Note*: Statistical analysis of PROM score changes between the sleep impaired (*n* = 517), and unimpaired (*n* = 622) arms by independent *t*‐test with significant differences denoted as *p < 0.050, **p < 0.010, ***p < 0.001.

Abbreviations: %, percentage; BPI, brief pain inventory; GAD‐7, generalized anxiety disorder‐7; *N*, number of participants; PGIC, patients' global impression of change; SD, standard deviation; SF‐MPQ‐2, short‐form McGill pain questionnaire‐2; SQS, single‐item sleep quality scale.

At 1‐ (sleep impaired *n* = 192, 37.14%; sleep unimpaired *n* = 187, 30.06%; *p* = 0.012), 6‐ (sleep impaired *n* = 175, 33.85%; sleep unimpaired *n* = 159, 25.56%; *p* = 0.002), and 12‐months (sleep impaired *n* = 132, 25.53%; sleep unimpaired *n* = 118, 18.97%; *p* = 0.008), those with sleep impairment were more likely to report a MCID according to the BPI pain severity subscale. There was no difference at 3‐months (sleep impaired *n* = 204, 39.46%; sleep unimpaired *n* = 225, 36.17%; *p* = 0.255).

Moreover, individuals who experienced a clinically significant improvement in sleep quality were more likely to report a MCID in pain severity at all time periods (*p* < 0.001).

Individuals with baseline sleep impairment were also more likely to report a MCID in sleep quality at 1‐ (sleep impaired *n* = 228, 44.10%; sleep unimpaired *n* = 81, 13.02%; *p* < 0.001), 3‐ (sleep impaired *n* = 197, 38.10%; sleep unimpaired *n* = 79, 12.70%; *p* < 0.001), 6‐ (sleep impaired *n* = 171, 33.08%; sleep unimpaired *n* = 69, 11.09%; *p* < 0.001), and 12‐months (sleep impaired *n* = 127, 24.56%; sleep unimpaired *n* = 52, 8.36%; *p* < 0.001).

### Oral morphine equivalents

Table 7 displays daily OME prescription doses for participants in both cohorts. The sleep impaired cohort had a mean OME dose of 255.9 mg/day, and there was no significant difference throughout (*p* > 0.050). The sleep unimpaired cohort had an OME dose of 91.1 mg/day at baseline that was significantly lower Median percentage change in daily OME dose at follow‐ups from baseline is displayed in Appendix F (Supporting Information). The reduction in median daily OME dose between the cohorts did not differ at any time‐point (*p* > 0.050).

**TABLE 7:** Median [IQR] oral morphine equivalent (OME) doses (mg/day) at baseline and follow‐up months 1, 3, 6, and 12 in the sleep impaired (*n* = 253), and unimpaired (*n* = 223) arms. Baseline defined at month 0.

Month	Sleep impaired (*n* = 253)		Sleep unimpaired (*n* = 223)	
	Median [IQR] OME dose (mg/day)	*p*‐value	Median [IQR] OME dose (mg/day)	*p*‐value
0	255.9 ± 916.9	–	91.1 ± 321.8	–
1	254.7 ± 916.6	1.000	87.8 ± 320.4	1.000
3	243.3 ± 892.5	1.000	85.3 ± 320.4	0.351
6	242.4 ± 892.6	0.987	82.0 ± 320.7	0.029*
12	234.5 ± 858.8	0.357	77.2 ± 308.3	0.146

*Note*: Statistical analysis using repeated measures analysis of variance for comparison of follow‐up OME dose against baseline, with significant differences denoted as *p < 0.050, **p < 0.010, ***p < 0.001.

Abbreviation: OME, oral morphine equivalent.

### Adverse events

**FIGURE 2:**
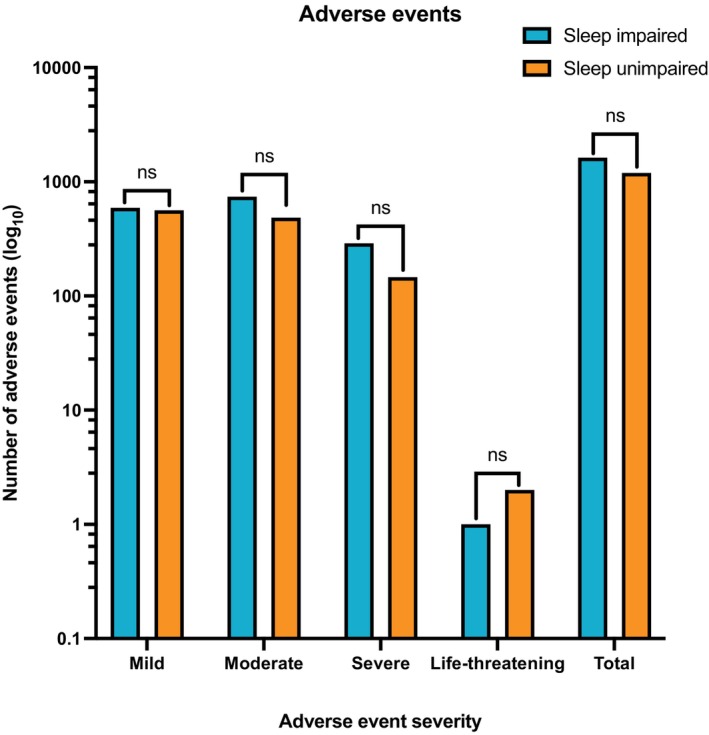
A bar graph showing adverse event frequency on a logarithmic_10_ scale separated by severity for the participants that experienced adverse events (*n* = 254) from baseline to 12 months, with the total number of adverse events displayed in the final column. Statistical analysis by Mann–Whitney *U* test for comparison of adverse event frequency between the arms. Log_10_, Logarithmic_10_; ns, Non‐significant.

A total of 2817 AEs were reported by 254 (22.3%) participants between both cohorts after initiation of CBMP treatment, of which 84.4% (*n* = 2378) were mild‐to‐moderate in severity. There was no difference in AE frequency between the two cohorts (Figure 2, Appendix G (Supporting Information)), with the sleep impaired arm reporting 1623 AEs (*n* = 145/5, 28.0%) and the sleep unimpaired group reporting 1194 AEs (*n* = 109/622, 18.5%; *p* = 0.197). The top 5 most common AEs were fatigue, dry mouth, lethargy, somnolence, and insomnia, as shown in Appendix H (Supporting Information).

### Univariate and multivariate analysis

A univariate analysis was conducted to assess for variables associated with a MCID change in pain severity^47^ at the 12‐month follow‐up (Appendix I (Supporting Information)). It found being underweight or obese (*p* < 0.050) was associated with a reduced odds of achieving this. However, it found a vapourised flower formulation either on its own or with sublingual oils (*p* < 0.001), a moderate‐to‐severe baseline BPI pain severity score (*p* < 0.050), and sleep impairment at baseline (*p* = 0.008) increased the likelihood of experiencing a MCID in pain severity.

A subsequent multivariate analysis (Appendix J (Supporting Information)) revealed an association between achieving the MCID in pain severity and moderate baseline BPI pain severity (OR = 2.734; 95% CI: 1.304–5.732; *p* = 0.008), severe baseline BPI pain severity (OR = 7.725; 95% CI: 3.547–16.826; *p* < 0.001), and being prescribed vapourised flower (OR = 3.061; 95% CI: 1.230–7.622; *p* = 0.016). Furthermore, an underweight or obese BMI maintained its association of decreasing these odds (*p* < 0.050). However, sleep impairment and the use of vapourised flower with sublingual oils no longer had a significant effect (*p* > 0.050). On the contrary, higher GAD‐7 scores were associated with a decreased likelihood of experiencing a MCID in pain severity (*p* < 0.050).

A univariate analysis to assess the variables that are associated with AE incidence (Appendix K (Supporting Information)) found sleep impairment (*p* < 0.001), BMI >40 kg/m^2^ (*p* = 0.004), and either cannabis naïvety (*p* < 0.001) or non‐current use (*p* = 0.007) increased the odds of reporting an AE. However, being male (*p* < 0.001), and the use of either vapourised flower alone (*p* = 0.015) or in combination with sublingual oils (*p* < 0.001) were associated with a lower likelihood of reporting an AE.

A further multivariate analysis to assess the relationship between these variables found being male continued to be associated with a lower AE incidence (OR = 0.556; 95% CI: 0.372–0.832; *p* = 0.004) (Appendix L (Supporting Information)), and sleep impairment continued to be associated with a higher likelihood (OR = 2.409; 95% CI: 1.603–3.621; *p* < 0.001).

## DISCUSSION

This UKMCR cohort study of CP patients with and without co‐morbid sleep impairment demonstrated improvements in validated pain‐specific and HRQoL PROMs at all follow‐ups in both cohorts except for SQS and EQ‐5D‐5L self‐care, and anxiety/depression in the sleep unimpaired arm. Furthermore, sleep impaired individuals demonstrated greater improvements in BPI pain severity, SQS, and PGIC scores at all follow‐ups than those sleep unimpaired. However, on multivariate analysis, those with sleep impairment were not more likely to report a clinically significant change in pain severity. There was no difference in AE frequency experienced in either cohort.

Following CBMP treatment initiation, the present study found improvements in mean pain‐specific PROM scores that are consistent with other comparable prospective open‐label observational studies.^31^, ^68^ A comprehensive review on phase I‐III RCTs of nabiximols,^69^ a licensed oromucosal THC and CBD spray,^70^ further supports the sustained benefits of CBMP treatment over 12 months. The review found that although not all patients benefitted from CBMP treatment, those that responded positively maintained the benefits up to 4 years^69^ in subsequent safety extension studies. At all time periods, over 1 in 5 participants reported a clinically significant improvement in pain severity. Except for month 3, those with sleep impairment at baseline were more likely to report a clinically significant difference.

The present study observed improvements in most HRQoL PROMs for all CP patients following CBMP treatment. A key HRQoL factor to consider is sleep. Whilst effective pain management improves HRQoL outcomes,^71^, ^72^, ^73^ CBMPs may also have a role in directly improving sleep quality. The consistent improvement in SQS score amongst the sleep impaired cohort up to 12‐months aligns with findings from RCTs investigating the relationship between CBMPs and sleep.^74^, ^75^ However, the CBMP formulations used in these studies include THC, CBD, and cannabinol, whereas the formulations in the present study did not always contain cannabinol. The RCTs also utilized the insomnia severity index (ISI) as a sleep measure, making it challenging to directly compare the SQS results in the present study against the ISI results in the RCTs. It is important to consider the clinical significance of these results. As expected, sleep impaired individuals were more likely to report clinical significance at all time periods. This was 44.10% at 1‐month, declining to 24.56% at 12‐months. This is an interesting outcome, considering improving sleep quality was not the primary goal of therapy. However, it is concerning that this decreases over time. This could be secondary to the baseline observation carried forward method used to handle missing data, but further studies should continue to assess whether tolerance develops to any effects CBMPs have on sleep.

Both cohorts displayed improvements in most PROMs at all follow‐up timepoints; however, observed improvements were greater in the sleep impaired cohort for BPI pain severity and SQS. Although the univariate analysis found sleep impairment was associated with increased odds of achieving the BPI pain severity MCID at 12‐months, this effect became non‐significant in the multivariate analysis, suggesting it was initially found to be significant due to its association with confounders (Appendix J (Supporting Information)). A possible confounder may be initial pain at baseline, in which the sleep impaired cohort had a higher baseline BPI pain severity score (*p* < 0.001, Appendix B (Supporting Information)) than the sleep unimpaired cohort. The multivariate analysis detected this was associated with a higher chance of achieving the MCID in BPI pain severity at 12‐months (Appendix J (Supporting Information)). This is corroborated by a study which observed clinical effects when treating moderate‐to‐severe symptoms but not for mild symptoms.^76^ This is further supported by Staquet et al., who reported that individuals with severe baseline pain experienced a more pronounced reduction in pain intensity compared to those with moderate baseline pain.^77^ Previous studies involving sleep deprivation in healthy participants have shown that diminished sleep quality can lower pain tolerance thresholds and impair pain modulation, resulting in increased cold, and pressure hyperalgesia.^36^, ^37^ Thus, sleep impairment may have exacerbated baseline pain severity in the sleep impaired cohort. In the present study, CBMP treatment may have contributed to improved sleep quality, leading to normalization of pain thresholds, and ultimately caused greater improvements in mean BPI pain severity scores than the sleep unimpaired cohort at all follow‐ups. This is supported by subgroup analysis in the present study which demonstrated that individuals who reported a clinically significant improvement in sleep quality, were also more likely to report improvements in pain severity.

There is significant heterogeneity in the CBMP formulations throughout the literature due to variations in the benefits and drawbacks of the different compounds, including THC, and CBD, as well as conflicting evidence regarding their co‐administration and potential synergistic effects. The present study prescribed a combination of THC, and CBD to most participants (*n* = 1087, 96.0%). Mitchell et al. found oral administration of THC alone caused a dose‐dependent decrease in allodynia but also resulted in a poorer side effect window due to its potency.^78^ Conversely, CBD alone demonstrated lower efficacy than THC; however, produced no side effects. Co‐administration of THC, and CBD in three different ratios (1:1, 1:8, and 1:80) displayed little to no synergy. In the present study, on multivariate analysis, the dose of CBD, and THC were not directly related to the likelihood of reporting a clinically significant improvement in pain severity or reporting adverse events. However, being prescribed dried flower was associated with a higher likelihood of reporting a clinically significant improvement in pain outcomes.

In the sleep impaired group, 17.8% (*n* = 45) of participants had a reduction in their OME, whilst 24.7% (*n* = 55) achieved this in the sleep unimpaired group. Other studies support this finding, showing a reduction in opioid consumption in CP patients associated with CBMPs.^79^, ^80^, ^81^ This effect may stem from CBMP action on CB1‐R, which forms heterodimers with μ‐opioid receptors at nociceptive terminals,^82^ thus activating μ‐opioid receptors and reducing opioid reliance. The effect size reporting in the present study may be lower than anticipated. Patients may have reduced their daily tablet intake without formally adjusting their prescribed dose, and this is particularly relevant for individuals who do not take opioids regularly but instead when required during acute exacerbations. Moreover, a large percentage of patients with available OME data reported prior or current cannabis use. These individuals may have therefore already tapered their opioid consumption prior to enrolment in the UKMCR.

Amongst 2817 reported AEs, 2378 (84.4%) were mild‐to‐moderate in severity. Consistent with previous CBMP studies, the most common AEs were fatigue, dry mouth, and lethargy.^30^, ^62^, ^83^ Notably, somnolence and insomnia were the fourth and fifth most reported AEs and may reflect symptoms of sleep impairment rather than direct consequences of CBMPs. However, to ensure improved compliance of patients self‐reporting AEs, these were not assessed to whether they were treatment‐related. This may explain the observed association between sleep impairment and increased odds of AE incidence (Appendixs K and L (Supporting Information)).

The present study's findings should be interpreted within the context of several limitations. The assessment of sleep impairment using SQS only captures symptoms over the past week, which may not reflect long‐term sleep impairment associated with CP, resulting in misclassification of patients who had poor sleep quality in the short‐term but do not typically experience sleep disturbances related to their CP. Alternative assessments, such as the ISI or Pittsburgh sleep quality index, consider symptoms over the past 2 weeks or past month, respectively, therefore may provide a more accurate representation of CP‐related sleep impairment. The study's observational design limits establishing a conclusive causal relationship between CBMP treatment and improvements in pain‐specific and HRQoL outcomes. Additionally, the absence of a control group makes it challenging to determine the extent to which observed improvements can be attributed to placebo effects. The placebo effect may be heightened in these patients due to media reporting of positive effects as well as the psychoactive effects of the medication.^84^, ^85^

Furthermore, potential selection biases arose from the utilization of data from a private medical cannabis registry. Since CBMPs are unlicenced for treating CP, only participants that were refractory to standard guideline treatments were referred to the clinic. Moreover, there was a higher proportion of previous or current cannabis users in the study (*n* = 773, 67.9%) compared to only 7.4% of adults in the England and Wales population that were reported to have used cannabis in the past year.^86^ Therefore, these selection biases limit the generalisability of the findings to the wider CP population.

## CONCLUSION

The results of this observational cohort study suggest an association between CBMP treatment and improvement in pain‐specific and HRQoL PROMs in CP patients with and without co‐morbid sleep impairment. Notably, those with co‐morbid sleep impairment were associated with greater improvements in BPI pain severity, SQS, and PGIC than those without. However, this finding was not confirmed on multivariate analysis. Reported sleep quality did improve across the cohort from baseline, and when present was also associated with improvements in pain severity, suggesting that the effects of CBMPs on sleep may provide additional benefits for individuals with chronic pain beyond affecting the transmission of nociceptive signals. At the onset of treatment, however, other variables may be better prognostic markers for response to CBMP treatment, such as severe pain or anxiety at baseline. With respect to clinical significance, 44.10% report an improvement in the sleep impaired cohort at 1‐months, declining to 24.56% at 12‐months. Despite being limited by its observational design, the present study can be used to inform future RCTs, in addition to current clinical practice.

## AUTHOR CONTRIBUTIONS

I.D., S.E., C.H., R.C., J.J.R., M.P., and M.H.S. designed the study. I.D., S.E., C.H., R.C., R.G., W.H., A.D.M., M.S., R.S., A.U., S.V., J.J.R., M.P. were all involved in data collection. I.D., S.E., and M.H.S were involved in data analysis and interpretation. However, all authors have had access to the study data. I.D., S.E., M.H.S. drafted the initial manuscript. All authors made critical edits and approved the final manuscript.

## FUNDING INFORMATION

There was no external or commercial funding associated with this paper. All authors have contributed to, and approved the final manuscript. The authors confirm that the PI for this paper is Mikael H Sodergren, and that he had direct clinical responsibility for patients.

## CONFLICT OF INTEREST STATEMENT

I.D. has no conflicts of interest to declare. S.E., C.H., R.C., R.G., W.H., A.D.M., M.S., R.S., A.U., S.V., J.J.R., M.P., and M.H.S are all either employed by or work on a consultant basis for Curaleaf Clinic, UK.

## PATIENT CONSENT STATEMENT

All participants completed written, informed consent prior to enrolment in the registry.

## Supporting information


Appendix S1.

## Data Availability

Data that support the findings of this study are available from the UK Medical Cannabis Registry. Restrictions apply to the availability of these data. Data specifications, and applications are available from the corresponding author.
