# Supplementary material for: UK medical cannabis registry: A clinical outcome analysis of medical cannabis therapy in chronic pain patients with and without co‐morbid sleep impairment
Source: Pain Pract. 2024 Nov 15;25(1):e13438. doi: 10.1111/papr.13438 (PMC11683519; doi:10.1111/papr.13438)
Supplement: Supplementary file 1 — Appendix S1. [file PAPR-25-0-s001.docx]

APPENDICES

*Appendix A. Table displaying frequency of recorded co-morbidities in the sleep impaired (n=517) and unimpaired (n=622) cohorts. Statistical analysis using chi-squared for comparisons between the cohorts, with significant differences denoted as *p<0.05, **p<0.01, ***p<0.001.*

| Co-morbidity | Sleep impaired (n=517) | Sleep unimpaired (n=622) | p-value |
| --- | --- | --- | --- |
|  | n (%) | n (%) |  |
| Myocardial infarction | 15 (2.9%) | 6 (1.0%) | 0.016* |
| Congestive heart failure | 5 (1.0%) | 5 (0.8%) | 0.769 |
| Peripheral vascular disease | 12 (2.3%) | 9 (1.4%) | 0.275 |
| Cerebrovascular accident | 21 (4.1%) | 19 (3.1%) | 0.358 |
| Dementia | 0 (0.0%) | 1 (0.2%) | 0.362 |
| Chronic obstructive pulmonary disease | 29 (5.6%) | 17 (2.7%) | 0.014* |
| Connective tissue disease | 97 (18.8%) | 114 (18.3%) | 0.851 |
| Peptic ulcer disease | 20 (3.9%) | 25 (4.0%) | 0.896 |
| Liver disease (mild) | 17 (3.3%) | 24 (3.9%) | 0.862 |
| Liver disease (moderate to severe) | 2 (0.4%) | 2 (0.3%) |  |
| Diabetes (uncomplicated) | 28 (5.4%) | 19 (3.1%) | 0.075 |
| Diabetes (end-organ damage) | 5 (1.0%) | 11 (1.8%) |  |
| Hemiplegia | 11 (2.1%) | 7 (1.1%) | 0.177 |
| Chronic kidney disease (moderate to severe) | 17 (3.3%) | 14 (2.3%) | 0.284 |
| Solid tumour (localised) | 29 (5.6%) | 17 (2.7%) | 0.023 |
| Solid tumour (metastatic) | 3 (0.6%) | 1 (0.2%) |  |
| Leukaemia | 2 (0.4%) | 1 (0.2%) | 0.459 |
| Lymphoma | 1 (0.2%) | 3 (0.5%) | 0.412 |
| Hypertension | 76 (14.7%) | 80 (12.9%) | 0.369 |
| Anxiety and/or depression | 211 (40.8%) | 214 (34.4%) | 0.026* |
| Arthritis | 183 (35.4%) | 195 (31.4%) | 0.149 |
| Epilepsy | 16 (3.1%) | 16 (2.6%) | 0.595 |
| Venous thromboembolism | 27 (5.2%) | 23 (3.4%) | 0.211 |

*Appendix B. Table displaying mean ± standard deviation baseline* *patient-reported outcome measure (PROM) scores of participants in the sleep impaired (n=517) and unimpaired (n=622) cohorts. Statistical analysis using independent t-tests for comparisons between the cohorts, with significant differences denoted as *p<0.05, **p<0.01, ***p<0.001. BPI: brief pain inventory; SF-MPQ-2: short-form McGill pain questionnaire-2; GAD-7: generalised anxiety disorder-7; SQS: single-item sleep quality scale;*

| PROMs | | Sleep impaired (n=517) | Sleep unimpaired (n=622) | p-value |
| --- | --- | --- | --- | --- |
|  |  | Mean ± SD | Mean ± SD |  |
| Pain-specific | BPI: pain severity | 6.44 ± 1.62 | 5.26 ± 1.75 | <0.001*** |
|  | BPI: Pain interference | 7.54 ± 1.86 | 5.63 ± 2.25 | <0.001*** |
|  | SF-MPQ-2 | 5.28 ± 1.97 | 3.82 ± 1.91 | <0.001*** |
| Health-related quality of life | GAD-7 | 9.19 ± 6.48 | 5.36 ± 5.23 | <0.001*** |
|  | SQS | 1.99 ± 1.09 | 6.05 ± 1.70 | <0.001*** |
|  | EQ-5D-5L: mobility | 3.13 ± 1.05 | 2.58 ± 1.16 | <0.001*** |
|  | EQ-5D-5L: selfcare | 2.41 ± 1.12 | 1.89 ± 1.00 | <0.001*** |
|  | EQ-5D-5L usual activities | 3.33 ± 1.08 | 2.70 ± 1.13 | <0.001*** |
|  | EQ-5D-5L: pain / discomfort | 4.06 ± 0.83 | 3.39 ± 0.91 | <0.001*** |
|  | EQ-5D-5L: anxiety / depression | 2.71 ± 1.23 | 2.02 ± 1.05 | <0.001*** |
|  | EQ-5D-5L: index value | 0.21 ± 0.30 | 0.45 ± 0.28 | <0.001*** |

*Appendix C. Paired mean percentage change ± standard deviation from baseline in patient-reported outcome measure (PROM) scores at 0, 1, 3, 6, and 12 months for the sleep impaired (n=517) and unimpaired (n=622) arms. Baseline PROM defined at month 0, with follow-up months at 1, 3, 6 and 12. Statistical analysis using repeated measures analysis of variance for comparison of follow-up percentage changes against baseline. Abbreviations: n: number of participants; SD: standard deviation; BPI: brief pain inventory; SF-MPQ-2: short-form McGill pain questionnaire-2; GAD-7: generalised anxiety disorder-7; SQS: single-item sleep quality scale; PGIC: patients’ global impression of change.*

| Patient related outcome measure | | Month | Sleep impaired | | | Sleep unimpaired | | |
| --- | --- | --- | --- | --- | --- | --- | --- | --- |
|  |  |  | n | Mean change in score ± SD (%) | p-value | n | Mean change in score ± SD (%) | p-value |
| Pain-specific | BPI: pain severity | 0 | 517 | 0.00 ± 0.00 | - | 622 | 0.00 ± 0.00 | - |
|  |  | 1 | 517 | -10.34 ± 24.03 | <0.001*** | 622 | -3.28 ± 41.77 | 0.503 |
|  |  | 3 | 517 | -11.91 ± 24.79 | <0.001*** | 622 | -7.19 ± 38.87 | <0.001*** |
|  |  | 6 | 517 | -11.52 ± 24.29 | <0.001*** | 622 | -6.58 ± 39.84 | <0.001*** |
|  |  | 12 | 517 | -8.90 ± 21.00 | <0.001*** | 622 | -3.34 ± 35.30 | 0.185 |
|  | BPI: pain interference | 0 | 517 | 0.00 ± 0.00 | - | 622 | 0.00 ± 0.00 | - |
|  |  | 1 | 517 | -15.23 ± 26.73 | <0.001*** | 622 | -1.61 ± 132.20 | 1.000 |
|  |  | 3 | 517 | -17.59 ± 29.85 | <0.001*** | 622 | -6.52 ± 112.62 | 1.000 |
|  |  | 6 | 517 | -15.25 ± 28.32 | <0.001*** | 622 | -2.42 ± 158.91 | 1.000 |
|  |  | 12 | 517 | -9.53 ± 26.03 | <0.001*** | 622 | -2.00 ± 142.51 | 1.000 |
|  | SF-MPQ-2 | 0 | 516 | 0.00 ± 0.00 | - | 620 | 0.00 ± 0.00 | - |
|  |  | 1 | 516 | -14.66 ± 30.24 | <0.001*** | 620 | -0.62 ± 150.58 | 1.000 |
|  |  | 3 | 516 | -14.35 ± 31.31 | <0.001*** | 620 | -2.99 ± 212.77 | 1.000 |
|  |  | 6 | 516 | -14.28 ± 31.84 | <0.001*** | 620 | -2.11 ± 207.38 | 1.000 |
|  |  | 12 | 516 | -9.83 ± 29.19 | <0.001*** | 620 | -0.08 ± 171.95 | 1.000 |

| Health-related quality of life | GAD-7 | 0 | 493 | 0.00 ± 0.00 | - | 570 | 0.00 ± 0.00 | - |
| --- | --- | --- | --- | --- | --- | --- | --- | --- |
|  |  | 1 | 493 | -18.05 ± 68.68 | <0.001*** | 570 | -3.00 ± 108.63 | 1.000 |
|  |  | 3 | 493 | -13.30 ± 67.29 | <0.001*** | 570 | -1.47 ± 98.13 | 1.000 |
|  |  | 6 | 493 | -10.18 ± 76.49 | 0.033* | 570 | -3.89 ± 76.78 | 1.000 |
|  |  | 12 | 493 | -3.03 ± 102.62 | 1.000 | 570 | 3.19 ± 96.83 | 1.000 |
|  | SQS | 0 | 453 | 0.00 ± 0.00 | - | 622 | 0.00 ± 0.00 | - |
|  |  | 1 | 453 | 109.68 ± 150.79 | <0.001*** | 622 | 7.90 ± 40.00 | <0.001*** |
|  |  | 3 | 453 | 94.33 ± 148.81 | <0.001*** | 622 | 8.75 ± 38.45 | <0.001*** |
|  |  | 6 | 453 | 81.68 ± 140.05 | <0.001*** | 622 | 7.46 ± 35.95 | <0.001*** |
|  |  | 12 | 453 | 60.41 ± 117.94 | <0.001*** | 622 | 5.18 ± 30.83 | <0.001*** |
|  | EQ-5D-5L: mobility | 0 | 517 | 0.00 ± 0.00 | - | 622 | 0.00 ± 0.00 | - |
|  |  | 1 | 517 | -5.73 ± 33.12 | <0.001*** | 622 | 0.86 ± 35.57 | 1.000 |
|  |  | 3 | 517 | -5.46 ± 32.55 | 0.002** | 622 | -0.61 ± 33.83 | 1.000 |
|  |  | 6 | 517 | -5.72 ± 29.35 | <0.001*** | 622 | -1.62 ± 31.77 | 1.000 |
|  |  | 12 | 517 | -2.93 ± 29.09 | 0.222 | 622 | 0.38 ± 28.61 | 1.000 |
|  | EQ-5D-5L: self-care | 0 | 517 | 0.00 ± 0.00 | - | 622 | 0.00 ± 0.00 | - |
|  |  | 1 | 517 | 0.02 ± 37.62 | 1.000 | 622 | 5.51 ± 41.47 | 0.010* |
|  |  | 3 | 517 | -1.37 ± 36.20 | 1.000 | 622 | 3.72 ± 35.52 | 0.093 |
|  |  | 6 | 517 | -0.27 ± 34.86 | 1.000 | 622 | 1.87 ± 35.16 | 1.000 |
|  |  | 12 | 517 | 0.38 ± 36.05 | 1.000 | 622 | 2.14 ± 30.24 | 0.779 |
|  | EQ-5D-5L: usual activities | 0 | 517 | 0.00 ± 0.00 | - | 622 | 0.00 ± 0.00 | - |
|  |  | 1 | 517 | -7.30 ± 44.26 | 0.002** | 622 | -4.32 ± 37.71 | 0.044* |
|  |  | 3 | 517 | -6.30 ± 38.62 | 0.002** | 622 | -2.70 ± 42.68 | 1.000 |
|  |  | 6 | 517 | -7.23 ± 37.92 | <0.001*** | 622 | -4.36 ± 31.86 | 0.007** |
|  |  | 12 | 517 | -3.31 ± 31.80 | 0.182 | 622 | -0.95 ± 35.65 | 1.000 |
|  | EQ-5D-5L: pain / discomfort | 0 | 517 | 0.00 ± 0.00 | - | 622 | 0.00 ± 0.00 | - |
|  |  | 1 | 517 | -13.52 ± 27.01 | <0.001*** | 622 | -8.55 ± 27.92 | <0.001*** |
|  |  | 3 | 517 | -13.28 ± 21.51 | <0.001*** | 622 | -9.54 ± 26.30 | <0.001*** |
|  |  | 6 | 517 | -12.03 ± 20.70 | <0.001*** | 622 | -9.31 ± 22.01 | <0.001*** |
|  |  | 12 | 517 | -9.88 ± 20.30 | <0.001*** | 622 | -5.43 ± 21.21 | <0.001*** |
|  | EQ-5D-5L: anxiety / depression | 0 | 517 | 0.00 ± 0.00 | - | 621 | 0.00 ± 0.00 | - |
|  |  | 1 | 517 | -8.42 ± 35.52 | <0.001*** | 621 | -1.23 ± 36.66 | 1.000 |
|  |  | 3 | 517 | -4.63 ± 40.49 | 0.096 | 621 | 2.95 ± 44.40 | 0.981 |
|  |  | 6 | 517 | -3.24 ± 36.61 | 0.445 | 621 | 1.66 ± 38.50 | 1.000 |
|  |  | 12 | 517 | -2.87 ± 29.07 | 0.254 | 621 | 3.44 ± 37.96 | 0.241 |
|  | EQ-5D-5L: index value | 0 | 517 | 0.21 ± 0.30 | - | 621 | 0.00 ± 0.00 | - |
|  |  | 1 | 517 | 547.81 ± 3379.27 | 0.003** | 621 | 219.87 ± 2621.07 | 0.370 |
|  |  | 3 | 517 | 285.74 ± 2265.85 | 0.043* | 621 | 106.12 ± 534.13 | <0.001*** |
|  |  | 6 | 517 | 400.39 ± 2828.22 | 0.014* | 621 | 108.72 ± 732.09 | 0.002** |
|  |  | 12 | 517 | 267.57 ± 1911.23 | 0.015* | 621 | 82.39 ± 649.65 (n=621) | 0.017* |

*Appendix D. Mean change ± standard deviation in patient-reported outcome measure (PROM) scores at follow-up months 1, 3, 6, and 12 compared to baseline. Statistical analysis of PROM score changes between the sleep impaired (n=517) and unimpaired (n=622) arms by independent t-test with significant differences denoted as *p<0.05, **p<0.01, ***p<0.001. Abbreviations: n: number of participants; SD: standard deviation; BPI: brief pain inventory; SF-MPQ-2: short-form McGill pain questionnaire-2; GAD-7: generalised anxiety disorder-7; SQS: single-item sleep quality scale;.*

| Patient-reported outcome measure | | Month | Sleep impaired (n=517) | Sleep unimpaired (n=622) | p-value |
| --- | --- | --- | --- | --- | --- |
|  |  |  | Mean change in score ± SD | Mean change in score ± SD |  |
| Pain- specific | BPI: pain severity | 1 | -0.74 ± 1.53 | -0.42 ± 1.47 | <0.001*** |
|  |  | 3 | -0.85 ± 1.57 | -0.56 ± 1.59 | 0.003** |
|  |  | 6 | -0.76 ± 1.50 | -0.50 ± 1.44 | 0.002** |
|  |  | 12 | -0.58 ± 1.27 | -0.32 ± 1.27 | <0.001*** |
|  | BPI: pain interference | 1 | -1.19 ± 1.83 | -0.64 ± 1.83 | <0.001*** |
|  |  | 3 | -1.39 ± 1.98 | -0.80 ± 2.00 | <0.001*** |
|  |  | 6 | -1.17 ± 1.93 | -0.67 ± 1.80 | <0.001*** |
|  |  | 12 | -0.76 ± 1.69 | -0.45 ± 1.62 | 0.002** |
|  | SF-MPQ-2 | 1 | -0.78 ± 1.41 | -0.43 ± 1.35 | <0.001*** |
|  |  | 3 | -0.78 ± 1.50 | -0.55 ± 1.40 | 0.010* |
|  |  | 6 | -0.79 ± 1.49 | -0.54 ± 1.38 | 0.003** |
|  |  | 12 | -0.54 ± 1.30 | -0.39 ± 1.25 | 0.053 |
| Health-related quality of life | GAD-7 | 1 | -2.42 ± 4.86 | -1.05 ± 3.98 | <0.001*** |
|  |  | 3 | -2.03 ± 4.79 | -0.77 ± 3.87 | <0.001*** |
|  |  | 6 | -1.77 ± 4.66 | -0.80 ± 3.41 | <0.001*** |
|  |  | 12 | -1.24 ± 4.00 | -0.48 ± 3.43 | <0.001*** |
|  | SQS | 1 | 2.27 ± 2.43 | 0.23 ± 2.03 | <0.001*** |
|  |  | 3 | 2.03 ± 2.49 | 0.30 ± 1.95 | <0.001*** |
|  |  | 6 | 1.82 ± 2.45 | 0.23 ± 1.85 | <0.001*** |
|  |  | 12 | 1.37 ± 2.20 | 0.15 ± 1.65 | <0.001*** |
|  | EQ-5D-5L: mobility | 1 | -0.27 ± 0.75 | -0.12 ± 0.66 | <0.001*** |
|  |  | 3 | -0.26 ± 0.74 | -0.13 ± 0.65 | 0.001** |
|  |  | 6 | -0.24 ± 0.69 | 0.15 ± 0.67 | 0.023* |
|  |  | 12 | -0.16 ± 0.61 | -0.07 ± 0.54 | 0.011* |
|  | EQ-5D-5L: self-care | 1 | -0.15 ± 0.74 | -0.01 ± 0.65 | <0.001*** |
|  |  | 3 | -0.16 ± 0.72 | -0.03 ± 0.59 | <0.001*** |
|  |  | 6 | -0.13 ± 0.67 | -0.06 ± 0.60 | 0.080 |
|  |  | 12 | -0.11 ± 0.63 | -0.04 ± 0.52 | 0.045* |
|  | EQ-5D-5L: usual activities | 1 | -0.44 ± 1.00 | -0.29 ± 0.86 | 0.008** |
|  |  | 3 | -0.37 ± 0.92 | -0.24 ± 0.89 | 0.009** |
|  |  | 6 | -0.37 ± 0.89 | -0.24 ± 0.78 | 0.005** |
|  |  | 12 | -0.23 ± 0.75 | -0.15 ± 0.71 | 0.041* |
|  | EQ-5D-5L: pain / discomfort | 1 | -0.63 ± 0.93 | -0.39 ± 0.84 | <0.001*** |
|  |  | 3 | -0.58 ± 0.87 | 0.41 ± 0.81 | <0.001*** |
|  |  | 6 | -0.53 ± 0.82 | -0.37 ± 0.74 | <0.001*** |
|  |  | 12 | -0.24 ± 0.80 | -0.24 ± 0.70 | <0.001*** |
|  | EQ-5D-5L: anxiety / depression | 1 | -0.37 ± 0.89 | -0.16 ± 0.78 | <0.001*** |
|  |  | 3 | -0.30 ± 0.91 | -0.10 ± 0.78 | <0.001*** |
|  |  | 6 | -0.23 ± 0.81 | -0.10 ± 0.72 | 0.003** |
|  |  | 12 | -0.18 ± 0.69 | -0.05 ± 0.65 | 0.001** |
|  | EQ-5D-5L: index value | 1 | 0.17 ± 0.25 | 0.09 ± 0.20 | <0.001*** |
|  |  | 3 | 0.15 ± 0.24 | 0.07 ± 0.19 | <0.001*** |
|  |  | 6 | 0.13 ± 0.23 | 0.07 ± 0.18 | <0.001*** |
|  |  | 12 | 0.10 ± 0.20 | 0.05 ± 0.17 | <0.001*** |

*Appendix E. Table showing mean ± standard deviation of patients’ global impression of change scores at follow-up months 1, 3, 6 and 12 for participants in the sleep impaired (n=458) and unimpaired (n=534) arms. Statistical analysis using independent t-tests for comparison between the two cohorts, with significance denoted as *p<0.050, **p<0.010, ***p<0.001. Abbreviations: n: number of participants; SD: standard deviation; PGIC: patients’ global impression of change.*

| PGIC month | Sleep impaired (n=458) | Sleep unimpaired (n=534) | p-value |
| --- | --- | --- | --- |
|  | Mean ± SD | Mean ± SD |  |
| 1 | 4.79 ± 1.58 | 5.16 ± 1.57 | <0.001*** |
| 3 | 5.02 ± 1.54 | 5.29 ± 1.54 | 0.006** |
| 6 | 5.15 ± 1.47 | 5.39 ± 1.48 | 0.012* |
| 12 | 5.20 ± 1.54 | 5.39 ± 1.51 | 0.047* |

*Appendix F. Median percentage change [IQR] in oral morphine equivalent (OME) doses at follow-up months 1, 3, 6 and 12 compared from baseline. Statistical analysis of OME dose changes between the sleep impaired (n=239) and unimpaired (n=214) cohorts by independent t-test, with significant with significant differences denoted as *p<0.050, **p<0.010, ***p<0.001. Abbreviations: OME: oral morphine equivalent.*

| Month | Sleep impaired (n=239) | Sleep unimpaired (n=214) | p-value |
| --- | --- | --- | --- |
|  | Median % change [IQR] in OME (mg/day) | Median % change [IQR] in OME (mg/day) |  |
| 1 | -1.1 ± 15.4 | -3.2 ± 37.9 | 0.423 |
| 3 | -12.6 ± 128.8 | -5.8 ± 41.1 | 0.455 |
| 6 | -13.5 ± 129.5 | -9.0 ± 44.9 | 0.626 |
| 12 | -21.4 ± 161.6 | -13.9 ± 84.4 | 0.266 |

*Appendix G. Table showing frequency of adverse events reported by participants in the sleep impaired (n=145) and unimpaired (n=109) cohorts, separated by severity with the final row displaying the total frequency. Statistical analysis using by Mann-Whitney U test for comparison of adverse event frequency between the arms.*

| Adverse event severity | Sleep impaired (n=145) | Sleep unimpaired (n=109) | p-value |
| --- | --- | --- | --- |
| Mild | 592 | 562 | 0.603 |
| Moderate | 740 | 484 | 0.056 |
| Severe | 290 | 146 | 0.063 |
| Life-threatening | 1 | 2 | 0.404 |
| Total | 1623 | 1194 | 0.197 |

*Appendix H. Table showing the frequency of adverse events reported by participants in the study (n=1139), separated by severity with the final column displaying the total frequency.*

| Adverse Events | Mild | Moderate | Severe | Life-threatening / Disabling | Total |
| --- | --- | --- | --- | --- | --- |
| Abdominal pain | 49 | 44 | 10 | 0 | 103 |
| Agitation | 0 | 3 | 1 | 0 | 4 |
| Amnesia | 16 | 10 | 2 | 0 | 28 |
| Anorexia | 22 | 30 | 7 | 0 | 59 |
| Anxiety | 2 | 7 | 4 | 0 | 13 |
| Arthralgia | 0 | 0 | 1 | 0 | 1 |
| Ataxia | 37 | 24 | 8 | 0 | 69 |
| Backpain | 0 | 2 | 1 | 0 | 3 |
| Belching | 0 | 1 | 0 | 0 | 1 |
| Bloating | 0 | 4 | 0 | 0 | 4 |
| Blurred vision | 32 | 28 | 14 | 0 | 74 |
| Chest pain | 0 | 0 | 2 | 0 | 2 |
| Cardiac chest pain | 0 | 1 | 0 | 0 | 1 |
| Cognitive disturbance | 35 | 34 | 7 | 0 | 76 |
| Colitis | 0 | 1 | 0 | 0 | 1 |
| Concentration impairment | 68 | 48 | 12 | 0 | 128 |
| Confusion | 31 | 16 | 5 | 0 | 52 |
| Constipation | 66 | 29 | 2 | 0 | 97 |
| Cough | 1 | 1 | 1 | 0 | 3 |
| COVID19 | 0 | 1 | 4 | 0 | 5 |
| Delirium | 18 | 8 | 2 | 0 | 28 |
| Delusions | 0 | 0 | 1 | 0 | 1 |
| Depression | 3 | 5 | 2 | 0 | 10 |
| Diarrhoea | 5 | 10 | 3 | 1 | 19 |
| Dizziness | 47 | 61 | 26 | 0 | 134 |
| Dry eye | 0 | 2 | 0 | 0 | 2 |
| Dry mouth | 156 | 31 | 0 | 0 | 187 |
| Dysgeusia | 17 | 11 | 6 | 0 | 34 |
| Dyspepsia | 51 | 24 | 13 | 0 | 88 |
| Dyspnoea | 0 | 1 | 0 | 0 | 1 |
| Dysuria | 1 | 0 | 0 | 0 | 1 |
| Facial pain | 0 | 2 | 0 | 0 | 2 |
| Fall | 12 | 7 | 1 | 0 | 20 |
| Fasciculations | 2 | 1 | 0 | 0 | 3 |
| Fatigue | 57 | 112 | 59 | 0 | 228 |
| Fever | 13 | 7 | 3 | 0 | 23 |
| Flatulence | 0 | 2 | 0 | 0 | 2 |
| Flu-like symptoms | 0 | 0 | 1 | 0 | 1 |
| Gastritis | 0 | 1 | 0 | 0 | 1 |
| Generalised muscle weakness | 24 | 30 | 32 | 0 | 86 |
| Headache | 46 | 59 | 46 | 0 | 151 |
| Hyperhidrosis | 0 | 1 | 0 | 0 | 1 |
| Hypersomnia | 0 | 1 | 0 | 0 | 1 |
| Hypertension | 0 | 0 | 1 | 0 | 1 |
| Increased appetite | 3 | 0 | 1 | 0 | 4 |
| Insomnia | 32 | 76 | 57 | 0 | 165 |
| Joint subluxation | 0 | 1 | 0 | 0 | 1 |
| Lethargy | 70 | 100 | 0 | 0 | 170 |
| Localised oedema | 1 | 0 | 0 | 0 | 1 |
| Mania | 0 | 1 | 0 | 0 | 1 |
| Oral mucositis | 0 | 0 | 1 | 0 | 1 |
| Muscle cramps | 0 | 1 | 0 | 0 | 1 |
| Myalgia | 0 | 2 | 0 | 0 | 2 |
| Nausea | 96 | 47 | 2 | 0 | 145 |
| Neck pain | 0 | 1 | 0 | 0 | 1 |
| Night terrors | 0 | 1 | 0 | 0 | 1 |
| Oedema (face) | 1 | 0 | 0 | 0 | 1 |
| Otitis (externa) | 0 | 1 | 0 | 0 | 1 |
| Pain | 0 | 4 | 9 | 0 | 13 |
| Pain in extremity | 1 | 0 | 2 | 0 | 3 |
| Palpitations | 2 | 3 | 1 | 0 | 6 |
| Paranoia | 0 | 0 | 1 | 0 | 1 |
| Paraesthesia | 0 | 1 | 2 | 0 | 3 |
| Peripheral sensory neuropathy | 0 | 3 | 0 | 0 | 3 |
| Pharyngitis | 0 | 42 | 5 | 0 | 47 |
| Post-COVID19 syndrome | 0 | 0 | 1 | 0 | 1 |
| Pruritus | 0 | 0 | 1 | 0 | 1 |
| Psychosis | 0 | 0 | 0 | 0 | 0 |
| Rash | 16 | 12 | 1 | 0 | 29 |
| Rhinorrhoea | 2 | 0 | 0 | 0 | 2 |
| Seizure | 0 | 1 | 2 | 0 | 3 |
| Sinus pain | 1 | 0 | 0 | 0 | 1 |
| Small Intestinal Bacterial Overgrowth | 0 | 0 | 1 | 0 | 1 |
| Somnolence | 0 | 144 | 22 | 0 | 166 |
| Sore throat | 0 | 0 | 1 | 0 | 1 |
| Spasticity | 8 | 11 | 15 | 0 | 34 |
| Stevens Johnson Syndrome | 0 | 0 | 1 | 0 | 1 |
| Suicidal ideation | 1 | 0 | 0 | 0 | 1 |
| Tremor | 13 | 12 | 5 | 0 | 30 |
| Upper respiratory infection | 0 | 23 | 5 | 0 | 28 |
| Urinary incontinence | 0 | 0 | 2 | 0 | 2 |
| Urinary tract infection | 0 | 19 | 1 | 1 | 21 |
| Uveitis | 0 | 1 | 0 | 0 | 1 |
| Vasovagal reaction | 0 | 0 | 1 | 1 | 2 |
| Vertigo | 38 | 39 | 15 | 0 | 92 |
| Vivid dreams | 0 | 0 | 0 | 0 | 0 |
| Vomiting | 23 | 10 | 2 | 0 | 35 |
| Weight gain | 0 | 1 | 1 | 0 | 2 |
| Weight loss | 35 | 7 | 0 | 0 | 42 |
| Wheezing | 0 | 0 | 1 | 0 | 1 |

Appendix I: *Table displaying the odds ratio (95% confidence interval) for the individual effect of variables on participants achieving the minimum clinically important difference in Brief Pain Inventory: pain severity at 12-month follow-up. Statistical analysis performed using a univariate logistic regression model, with significance denoted as *p<0.050, **p<0.010, ***p<0.001. BPI: brief pain inventory; CBD – cannabidiol; CBMP – cannabis-based medicinal product; GAD-7: generalised anxiety disorder-7; THC – tetrahydrocannabinol; SQS: single-item sleep quality scale.*

| Variable | n | Odds ratio (95% CI) | p-value |
| --- | --- | --- | --- |
| Gender |  |  |  |
| Female | 525 | - |  |
| Male | 614 | 0.845 (0.569 – 1.253) | 0.402 |
| Age (years) |  |  |  |
| 18 – 30 | 147 | - |  |
| 31 – 40 | 282 | 1.051 (0.546 – 2.023) | 0.881 |
| 41 – 50 | 326 | 1.424 (0.759 – 2.672) | 0.271 |
| 51 – 60 | 199 | 1.680 (0.601 – 4.698) | 0.323 |
| 61 – 70 | 105 | 1.701 (0.560 – 5.163) | 0.348 |
| > 70 | 80 | 0.817 (0.222 – 3.016) | 0.762 |
| Body mass index (kg/m^2^) |  |  |  |
| < 18.5 | 43 | 0.222 (0.060 – 0.818) | 0.024* |
| 18.5 – 24.9 | 388 | - |  |
| 25.0 – 29.9 | 327 | 0.679 (0.439 – 1.051) | 0.082 |
| 30.0 – 39.9 | 235 | 0.597 (0.369 – 0.967) | 0.036* |
| > 40.0 | 53 | 0.839 (0.366 – 1.923) | 0.677 |
| Charlson co-morbidity index |  |  |  |
| None (0) | 454 | - |  |
| Mild (1–2) | 218 | 0.985 (0.597 – 1.623) | 0.951 |
| Moderate (3–4) | 94 | 1.074 (0.432 – 2.672) | 0.878 |
| Severe (> 5) | 373 | 1.121 (0.432 – 2.587) | 0.790 |
| Cannabis status |  |  |  |
| Never used | 366 | - |  |
| Ex-user | 165 | 1.196 (0.632 – 2.262) | 0.583 |
| Current user | 606 | 1.309 (1.791 – 2.166) | 0.295 |
| CBMP route of administration |  |  |  |
| Sublingual & oral formulations only | 342 | - |  |
| Vapourised flower only | 253 | 2.234 (1.487 – 3.357) | <0.001*** |
| Both | 538 | 1.842 (1.286 – 2.639) | <0.001*** |
| Total CBD dosage |  |  |  |
| None (0 mg/day) | 31 | - |  |
| < median dose (20.5 mg/day) | 550 | 1.949 (0.581 – 6.542) | 0.280 |
| > median dose (20.5 mg/day) | 551 | 3.523 (1.056 – 11.760) | 0.041* |
| Total THC dosage |  |  |  |
| None (0 mg/day) | 14 | - |  |
| < median dose (112.0 mg/day) | 560 | 0.638 (0.174 – 2.336) | 0.638 |
| > median dose (112.0 mg/day) | 558 | 1.513 (0.417 – 5.494) | 0.680 |
| BPI: pain severity baseline score |  |  |  |
| None to mild (0–4) | 185 | - |  |
| Moderate (5–6) | 333 | 1.717 (1.053 – 2.800) | 0.030* |
| Severe (7–10) | 313 | 2.430 (1.501 – 3.934) | <0.001*** |
| GAD-7 baseline score |  |  |  |
| None (0–4) | 490 | - |  |
| Mild (5–9) | 294 | 0.961 (0.678 – 1.363) | 0.824 |
| Moderate (10–14) | 182 | 1.036 (0.691 – 1.554) | 0.863 |
| Severe (> 15) | 173 | 0.845 (0.549 – 1.300) | 0.444 |
| SQS baseline score |  |  |  |
| Sleep unimpaired (> 4) | 622 | - |  |
| Sleep impaired (< 3) | 517 | 1.464 (1.105 – 1.941) | 0.008** |

Appendix J: *Table displaying the odds ratio (95% confidence interval) for the effect of variables on participants achieving the minimum clinically important difference in Brief Pain Inventory pain severity at 12-month follow-up. Statistical analysis performed using a multivariate logistic regression model, with significance denoted as *p<0.050, **p<0.010, ***p<0.001. BPI: brief pain inventory; CBD – cannabidiol; CBMP – cannabis-based medicinal product; GAD-7: generalised anxiety disorder-7; THC – tetrahydrocannabinol; SQS: single-item sleep quality scale;*

| Variable | n | Odds ratio (95% CI) | p-value |
| --- | --- | --- | --- |
| Gender |  |  |  |
| Female | 341 | - |  |
| Male | 422 | 0.845 (0.571 – 1.253) | 0.402 |
| Age (years) |  |  |  |
| 18 – 30 | 100 | - |  |
| 31 – 40 | 193 | 1.005 (0.521 – 1.939) | 0.989 |
| 41 – 50 | 222 | 1.429 (0.766 – 2.666) | 0.262 |
| 51 – 60 | 133 | 1.812 (0.934 – 3.517) | 0.079 |
| 61 – 70 | 72 | 1.843 (0.817 – 4.158) | 0.141 |
| > 70 | 43 | 0.862 (0.275 – 2.698) | 0.799 |
| Body mass index (kg/m^2^) |  |  |  |
| < 18.5 | 30 | 0.208 (0.056 – 0.777) | 0.019* |
| 18.5 – 24.9 | 289 | - |  |
| 25.0 – 29.9 | 233 | 0.664 (0.429 – 1.028) | 0.066 |
| 30.0 – 39.9 | 173 | 0.583 (0.360 – 0.946) | 0.029* |
| > 40.0 | 38 | 0.812 (0.353 – 1.867) | 0.402 |
| Cannabis status |  |  |  |
| Never used | 426 | - |  |
| Ex-user | 110 | 1.192 (0.628 – 2.262) | 0.591 |
| Current user | 227 | 1.264 (0.750 – 2.130) | 0.380 |
| CBMP route of administration |  |  |  |
| Sublingual & oral formulations only | 211 | - |  |
| Vapourised flower only | 178 | 1.452 (0.693 – 3.041) | 0.323 |
| Both | 374 | 0.936 (0.494 – 1.775) | 0.839 |
| Total CBD dosage |  |  |  |
| None (0 mg/day) | 22 | - |  |
| < median dose (20.5 mg/day) | 353 | 1.168 (0.321 – 4.253) | 0.814 |
| > median dose (20.5 mg/day) | 388 | 2.110 (0.573 – 7.762) | 0.261 |
| Total THC dosage |  |  |  |
| None (0 mg/day) | 9 | - |  |
| < median dose (112.0 mg/day) | 362 | 0.950 (0.172 – 5.236) | 0.953 |
| > median dose (112.0 mg/day) | 392 | 1.823 (0.312 – 10.642) | 0.505 |
| BPI: severity baseline score |  |  |  |
| None to mild (0–4) | 168 | - |  |
| Moderate (5–6) | 310 | 1.891 (1.099 – 3.254) | 0.021* |
| Severe (7–10) | 285 | 2.823 (1.607 – 4.960) | <0.001*** |
| GAD-7 baseline score |  |  |  |
| None (0–4) | 325 | - |  |
| Mild (5–9) | 196 | 0.849 (0.544 – 1.325) | 0.471 |
| Moderate (10–14) | 117 | 0.529 (0.302 – 0.926) | 0.026* |
| Severe (> 15) | 125 | 0.440 (0.246 – 0.786) | 0.006** |
| SQS baseline score |  |  |  |
| Sleep unimpaired (> 4) | 403 | - |  |
| Sleep impaired (< 3) | 360 | 1.427 (0.960 – 2.121) | 0.079 |

*Appendix K. Table displaying the odds ratio (95% confidence interval) for the effect of variables individually on participants experiencing an adverse event. Statistical analysis performed using a univariate logistic regression model, with significance denoted as *p<0.050, **p<0.010, ***p<0.001. BPI: brief pain inventory; CBD – cannabidiol; CBMP – cannabis-based medicinal product; GAD-7: generalised anxiety disorder-7; THC – tetrahydrocannabinol; SQS: single-item sleep quality scale.*

| Variable | n | Odds ratio (95% CI) | p-value |
| --- | --- | --- | --- |
| Gender |  |  |  |
| Female | 525 | - |  |
| Male | 614 | 0.430 (0.323 – 0.573) | <0.001*** |
| Age (years) |  |  |  |
| 18 – 30 | 147 | - |  |
| 31 – 40 | 282 | 0.751 (0.462 – 1.223) | 0.250 |
| 41 – 50 | 326 | 0.689 (0.427 – 1.113) | 0.128 |
| 51 – 60 | 199 | 1.238 (0.755 – 2.030) | 0.398 |
| 61 – 70 | 105 | 1.734 (0.994 – 3.024) | 0.052 |
| > 70 | 80 | 1.261 (0.676 – 2.350) | 0.466 |
| Body mass index (kg/m^2^) |  |  |  |
| < 18.5 | 43 | 1.263 (0.611 – 2.613) | 0.529 |
| 18.5 – 24.9 | 388 | - |  |
| 25.0 – 29.9 | 327 | 0.894 (0.621 – 1.289) | 0.549 |
| 30.0 – 39.9 | 235 | 1.204 (0.821 – 1.766) | 0.342 |
| > 40.0 | 53 | 2.412 (1.321 – 4.401) | 0.004** |
| Charlson co-morbidity index |  |  |  |
| None (0) | 454 | - |  |
| Mild (1–2) | 218 | 1.558 (1.047 – 2.318) | 0.029* |
| Moderate (3–4) | 94 | 1.431 (0.830 – 2.466) | 0.197 |
| Severe (> 5) | 373 | 1.949 (1.395 – 2.722) | <0.001*** |
| Cannabis status |  |  |  |
| Current user | 608 | - |  |
| Ex-user | 165 | 1.763 (1.165 – 2.670) | 0.007** |
| Never used | 366 | 2.506 (1.839 – 3.415) | <0.001*** |
| CBMP route of administration |  |  |  |
| Sublingual & oral formulations only | 342 | - |  |
| Vapourised flower only | 253 | 0.620 (0.421 – 0.912) | 0.015* |
| Both | 538 | 0.581 (0.423 – 0.798) | <0.001*** |
| Total CBD dosage |  |  |  |
| None (0 mg/day) | 31 | - |  |
| < median dose (20.5 mg/day) | 550 | 2.495 (0.745 – 8.350) | 0.138 |
| > median dose (20.5 mg/day) | 551 | 2.970 (0.889 – 9.924) | 0.077 |
| Total THC dosage |  |  |  |
| None (0 mg/day) | 14 | - |  |
| < median dose (112.0 mg/day) | 560 | 1.246 (0.343 – 4.528) | 0.739 |
| > median dose (112.0 mg/day) | 558 | 0.870 (0.239 – 3.173) | 0.833 |
| SQS baseline score |  |  |  |
| Sleep unimpaired (> 4) | 622 | - |  |
| Sleep impaired (< 3) | 517 | 1.834 (1.384 – 2.432) | <0.001*** |

*Appendix L. Table displaying the odds ratio (95% confidence interval) for the effect of variables on participants experiencing an adverse event. Statistical analysis performed using a multivariate logistic regression model, with significance denoted as *p<0.050, **p<0.010, ***p<0.001. BPI: brief pain inventory; CBD – cannabidiol; CBMP – cannabis-based medicinal product; GAD-7: generalised anxiety disorder-7; THC – tetrahydrocannabinol; SQS: single-item sleep quality scale.*

| Variable | n | Odds ratio (95% CI) | p-value |
| --- | --- | --- | --- |
| Gender |  |  |  |
| Female | 341 | - |  |
| Male | 422 | 0.556 (0.372 – 0.832) | 0.004** |
| Age (years) |  |  |  |
| 18 – 30 | 100 | - |  |
| 31 – 40 | 193 | 0.963 (0.509 – 1.820) | 0.907 |
| 41 – 50 | 222 | 0.744 (0.392 – 1.410) | 0.364 |
| 51 – 60 | 133 | 0.872 (0.310 – 2.454) | 0.796 |
| 61 – 70 | 72 | 0.960 (0.318 – 2.895) | 0.942 |
| > 70 | 43 | 0.845 (0.274 – 2.610) | 0.770 |
| Body mass index (kg/m^2^) |  |  |  |
| < 18.5 | 30 | 1.379 (0.552 – 3.440) | 0.491 |
| 18.5 – 24.9 | 289 | - |  |
| 25.0 – 29.9 | 233 | 1.138 (0.714 – 1.815) | 0.587 |
| 30.0 – 39.9 | 173 | 1.546 (0961 – 2.487) | 0.073 |
| > 40.0 | 38 | 1.788 (0.814 – 3.927) | 0.148 |
| Charlson co-morbidity index |  |  |  |
| None (0) | 306 | - |  |
| Mild (1–2) | 149 | 1.249 (0.752 – 2.074) | 0.391 |
| Moderate (3–4) | 50 | 1.045 (0.416 – 2.626) | 0.926 |
| Severe (> 5) | 258 | 1.413 (0.597 – 3.346) | 0.431 |
| Cannabis status |  |  |  |
| Current user | 426 | - |  |
| Ex-user | 110 | 1.609 (0.932 – 2.775) | 0.088 |
| Never used | 227 | 1.567 (0.932 – 2.633) | 0.090 |
| CBMP route of administration |  |  |  |
| Sublingual & oral formulations only | 211 | - |  |
| Vapourised flower only | 178 | 1.643 (0.813 – 3.323) | 0.167 |
| Both | 374 | 1.090 (0.617 – 1.925) | 0.767 |
| Total CBD dosage |  |  |  |
| None (0 mg/day) | 22 | - |  |
| < median dose (20.5 mg/day) | 353 | 3.550 (0.678 – 16.417) | 0.105 |
| > median dose (20.5 mg/day) | 388 | 4.596 (0.985 – 21.450) | 0.052 |
| Total THC dosage |  |  |  |
| None (0 mg/day) | 9 | - |  |
| < median dose (112.0 mg/day) | 362 | 1.445 (0.269 – 7.774) | 0.668 |
| > median dose (112.0 mg/day) | 392 | 1.234 (0.127 – 7.028) | 0.764 |
| SQS baseline score |  |  |  |
| Sleep unimpaired (> 4) | 403 | - |  |
| Sleep impaired (< 3) | 360 | 2.409 (1.603 – 3.621) | <0.001*** |
